# Leading the Challenge: Leader Support Modifies the Effect of Role Ambiguity on Engagement and Extra-Role Behaviors in Public Employees

**DOI:** 10.3390/ijerph18168408

**Published:** 2021-08-09

**Authors:** Ana Martínez-Díaz, Miguel A. Mañas-Rodríguez, Pedro A. Díaz-Fúnez, José M. Aguilar-Parra

**Affiliations:** 1 IPTORA Research Team, University of Almería, 04120 Almería, Spain; amd479@inlumine.ual.es (A.M.-D.); marodrig@ual.es (M.A.M.-R.); 2 Hum-878 Research Team, Health Research Centre, Department of Psychology, University of Almería, 04120 Almería, Spain; jmaguilar@ual.es

**Keywords:** role ambiguity, leader support, engagement, extra-role performance behaviors, moderate mediation model, positive psychology

## Abstract

The assumption of new challenges and services to provide, and the evolution of new technologies in public administration, give employees an important perception of ambiguity when carrying out their work. Role ambiguity has been conceptualized as one of the main impeding demands at work with negative consequences. The objective of the present study is to analyze the moderating effect of the support by the department head in the negative influence of the role ambiguity on the engagement and the extra-role performance behaviors of the employees. The hypothesis is proposed that the support of the department head will mean the transformation of role ambiguity into a challenging job demand with positive results. A total of 315 public employees with administrative staff have participated in this study. Results confirmed that the support of the leader moderates the effects of role ambiguity. The inclusion of this variable as a moderator transforms the influence of role ambiguity on the employees’ engagement into a positive one and reduces their negative effect on extra-role performance behaviors. These results reinforce the role of leader support as a protective element against job demands in public administrations. Theoretical and practical implications and future lines of research are discussed at the end of the work.

## 1. Introduction

In recent decades, public services have undergone a great transformation with continuous reforms of their structures, increased bureaucratization of systems and processes, the appearance of new demands such as the great threat to public health due to coronavirus (COVID-19), new services to be provided, and the need for new skills and capacities in employees for the performance of their duties [1,2]. These changes aim to adapt to new ways of working and increase efficiency, profitability, and performance in the public sector [3]. Nevertheless, all these changes are having negative consequences on public employees since they are in charge of satisfying the growing demands of citizens in a context of constant change and with fewer resources [4,5].

This new context in public administration leads to an increase in the lack of clarity in the functions they perform, reflecting a negative impact on public employees’ emotions [6]. Still, it is not clear that it may affect their performance. Role ambiguity is one of the most researched job demands, showing important negative consequences in the work context. This variable is characterized by increased uncertainty in employees about acting when facing a situation with insufficient and unclear information about their objectives, responsibilities, and tasks [7]. When clear goals are not perceived, employees show less engagement in the organization. Consequently, they put less effort into carrying out performance behaviors that go beyond what is required by their job [8].

Even though job demands have traditionally been approached as factors that affect employees in a negative process of deterioration in health, recent theories have proposed that they can play a positive role within the motivational process [9]. Previous studies such as Flinchbauh, Luth, and Li [10] have already shown that demands do not necessarily have to be factors that increase tension; how they are perceived will depend on other contextual factors. However, research is still scarce in the public sector; in role ambiguity, the leader’s supportive behavior may offset the negative health effects [11]. A context where the leader offers support allows employees to feel the work is a challenge and become more involved in the tasks and objectives of the organization [12,13] and increases the probability of achieving success in the business environment [14]. Therefore, this study provides interesting guidelines in the public sphere on the exchange of support by the department head that promotes the sense of competence, decision-making skills, loyalty, engagement, and performance of employees [15].

This work contributes to the debate of the capacity of leader support to reduce the negative effects of job demands and interpret this activity’s nature in public services. First, this article looks at how role ambiguity in public services can be a positive resource that increases employees engagement. It then addresses the issue of stress job demands in public services by examining three processes: (1) its initial negative influence on extra-role performance behaviors, (2) how this negative influence is due, in part, to the reduction it generates in employee engagement, and (3) how adequate support from leaders can be the key element that reduces and makes the effect of ambiguity positive. This study will provide information and guidance to public administrations in formulating positive management strategies for their work teams and training those responsible for these work teams, developing positive work environments. 

### Theoretical Framework 

One of the most relevant models in recent years when analyzing the influence of organizational contexts on individuals is the theory of job demands and resources [9]. From this theory, job demands and resources are described as the starting point of two different processes. The job demands originate a deterioration of the health, with negative influences on the employees, causing their emotional deterioration and making it difficult for organizations to achieve their objectives. If job demands persist and are not reduced, they can lead to serious health problems and undermine employee engagement. On the other hand, labor resources are the starting point of a motivational process, with positive consequences at the motivational and behavioral level for employees, making it easier for organizations to meet their objectives [16].

From the JD–R theory, job demands are conceptualized as physical and psychological aspects associated with work that require effort for the individual [17]. Studies based on this theory have revealed that job demands, such as role ambiguity, can lead to exhaustion, disconnection, low job satisfaction, and deterioration in employee health [18], while labor resources can generate a motivating process that leads to related learning, job satisfaction, and work and organizational engagement [19].

Role ambiguity is one of the main job demands due to its frequent presence in the work context [20,21]. This occurs when there are contradictions about the functions that a collaborator has to carry out, how they have to carry out those functions, or a lack of knowledge about the objectives and responsibilities pursued with them [22]. Since the introduction of the concept of role ambiguity by Kahn et al. [23], research has consistently demonstrated its negative influence on achieving job objectives, limiting the capacity and effectiveness of employees in their jobs [8].

Prior studies have revealed that negative job features such as stressful job demands at work could inhibit proactivity at work [24]. This study focuses on the impacts of one particular job stressor, role ambiguity, on proactive behaviors. Extra-role performance behaviors can be defined as voluntary behaviors not included in the formal requirements of the job, and therefore, involve performing tasks at a level beyond what is required or expected [25,26]. These types of behaviors provide organizations with a better capacity to respond and adapt to unexpected events and difficulties that have arisen [27]. In the literature on public organizations, it is well known that they have more ambiguous elements and that these negatively influence the enthusiasm, motivation, and performance of public employees [28], but what happens with the extra-role behavior of these organizations? Caillier [29] points out that role ambiguity in a work context reduces voluntary behaviors in the organization. Lack of clarity reduces the probability that individuals try to carry out behaviors beyond what is required of them if they already experience difficulties developing the essential functions of their job, which influences job performance [30]. For this reason, the following hypothesis is proposed.

**Hypothesis** **1.** *The degree of role ambiguity will show a significant negative influence on the propensity to carry out extra-role performance behaviors in the workplace.*

In line with the previous theoretical extensions on the JD–R model, it is plausible to suggest that stressful job demands, such as role ambiguity, may decrease employees’ level of work engagement, which in turn produces negative performance-related outcomes [31]. Engagement is most often defined as “a positive, job-related state of mind that is characterized by vigor, dedication, and absorption” ([32], p. 74). When employees’ perception of engagement is stimulated, it allows them to develop positive attitudes towards work and generate a competitive advantage for the organization [33]. Various studies have shown that role clarity directly and positively affects engagement since a better understanding of an employee’s performance expectations makes them feel more responsible and engaged in their work [34,35]. However, when confronted with such hindrances as incompatible or unclear role demands, employees may experience negative emotions and tend to adopt a passive coping style [36,37]. Based on this, the following hypothesis is proposed.

**Hypothesis** **2.** *Role ambiguity will show a significant negative influence on engagement.*

The relationship between role ambiguity, engagement, and employee extra-role performance behaviors can be delineated by drawing on the extensions of the JD–R model [9,16,17]. This has confirmed important distinctions between job demands regarding how employees perceive them and that when this distinction is considered, significant links between job demands and engagement may occur [38]. On the other hand, employees can perceive some job demands as necessary to promote personal growth and development and thus enhance their work engagement [39], while stressful job demands, such as role ambiguity, tend to deplete employees’ energy, tax their capacities, and decrease their work engagement [36]. Therefore, the following hypothesis is suggested.

**Hypothesis** **3.** *The degree of role ambiguity will influence the propensity to carry out extra-role performance behaviors in the workplace through the engagement of employees.*

In recent research from the JD–R theory, authors such as Harms et al. [40] propose that one way to reduce or control existing demands in the workplace is to provide employees with new resources from the organization. The distinction between job demands perceived as an obstacle or as a challenge has been raised [10]. The former inhibits personal growth and the achievement of workers’ objectives [41]. By contrast, challenge demands are defined as aspects that require effort but potentially promote personal growth and the perception of employees’ effectiveness [42]. Despite the abundant theoretical and empirical evidence supporting the negative impact of job demands [43,44], recent studies are also engaging in its possible positive effects [45,46]. Still, few studies have focused on the context of public administration.

One of the labor resources that has received the most attention in the last 3 decades is transformational leadership [47]. First proposed by Burns [48] and developed by Bass [49], this leadership style is geared towards meeting the needs of followers, improving group cohesion, and influencing employee confidence [50]. Authors such as Rafferty and Griffin [51] affirm that it is vital to support the efforts in a demanding organizational context, encourage autonomy, and train employees to assume greater responsibilities in the work environment. Although at first the behavior of leaders may also be affected by the level of context stress, a workplace where support is provided will show beneficial results for both employees and the organization by fostering respect, trust, cooperation, and emotional support [52]. Therefore, the following hypothesis is proposed.

**Hypothesis** **4.** *Role ambiguity will show a significant influence of negative signs on leader support.*

It has been pointed out that role ambiguity plays an essential role in extra-role performance behavior [53]. Some studies have shown that employees will carry out more voluntary behaviors after receiving support from the leader in highly ambiguous situations [2,29,54]. This exposes that employees in ambiguous contexts will put more effort at work and perform behaviors beyond what is formally established when there are supportive behaviors [55]. On the contrary, employees who do not receive any support limit themselves to fulfilling pre-established tasks [56]. Consequently, the different transformational leadership studies show the positive effects on the development of workers’ resources [51,57]. Based on this, the following hypothesis is proposed.

**Hypothesis** **5.** *The degree of role ambiguity will influence the propensity to carry out extra-role performance behaviors in the workplace through leader support.*

Proposition 4 of the JD–R theory establishes that labor resources positively influence worker engagement when job demands are high [9]. Different studies such as Martínez [58] or Van den Broeck et al. [36] have shown the importance of job resources as predictors of engagement when the work context requires high demands. In this sense, when role ambiguity is high, employees who perceive support from the leader will develop higher levels of engagement by challenging them to more effectively integrate their capabilities in the work context [42]. This highly ambiguous situation will produce higher levels of engagement in employees due to the leader’s supportive behaviors in the work context [59]. Therefore, the following hypothesis is proposed.

**Hypothesis** **6.** *The effect of role ambiguity on engagement is moderated by the degree of support from the department head.*

Previous studies have delved into the effect of leader support on stress. For example, in a study with soldiers, Britt et al. [60] found that leader support protects individuals from the negative influence of stressful events. However, these authors concluded in their work that the supervisor’s support is effective, especially in a high level of stress. The present study finds protection against stress and produces positive effects on the employees’ extra-role performance behaviors. Therefore, it is interesting to study the effects of leader support on stress in different contexts and on both personal and organizational aspects [61], and the subsequent hypothesis is proposed.

**Hypothesis** **7.** *The mediating process of engagement on the influence of role ambiguity on extra-role performance behaviors will depend on supportive behaviors that the leader will carry out.*

**Figure 1:**
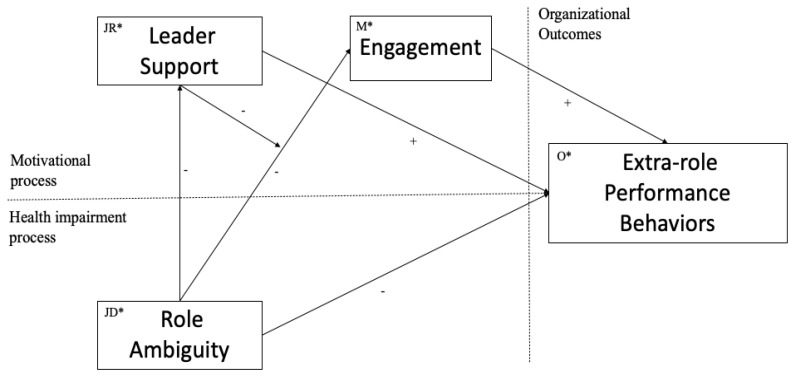
Research model. JD* = job demands; JR* = job resources; M* = motivation; O* = outcomes.

Moreover, previous studies do not clarify whether there is a possibility that that one variable acts as a mediator and a moderator at the same time in a relationship model [62]. This has added to the confusion about the concept of moderation [63]. Following the opinion of experts on the subject [64], the present manuscript proposes that, indeed, a variable can adopt this double role within a model (see Figure 1).

For all the above, this work proposes to analyze the conditions in which role ambiguity is related to extra-role performance behaviors of employees through engagement and leadership. This proposal examines how role ambiguity and engagement are affected by the moderating effect of support from the leader. We hope that employees who receive support from the leader will transform this situation into a challenge when they work with high ambiguity. Furthermore, this research extends the model of job demands and resources by exploring the influence of a labor resource when transforming the consequences of job demands into positives.

## 2. Materials and Methods

### 2.1. Procedure

This study was approved by the Ethics Committee of the University of Almería. The data were collected from the administrative staff of employees of a public organization located in Spain. The research team contacted and explained the purpose of the project to the management of the public administration. Once they agreed to collaborate, workers in each department were informed about the study’s relevance, achieving participation and ensuring confidentiality and anonymity. Participants signed informed consent and filled out the online questionnaires during their workday. In case they had any questions, some members of the investigation team were available to solve them. 

### 2.2. Sample

The sample consisted of employees of administrative staff in public administration located in Spain. This research collects data through a questionnaire that employees received via email. A total of 390 questionnaires were distributed, trying to cover all the employees of the organization. Of these, 315 responses were considered valid among the 357 questionnaires received, for a response rate of 80.77%. This sample is distributed based on the following variables (See Table 1). 

### 2.3. Measures

#### 2.3.1. Leader Support

This dimension was assessed using the Rafferty and Griffin [51] transformational leadership questionnaire. The questionnaire to evaluate this dimension is made up of 3 items (e.g., “Our leader thinks about our intellectual needs”). A Likert-type response scale was used, with values ranging from 0 (strongly disagree) to 6 (strongly agree).

#### 2.3.2. Engagement

The engagement has been measured with the Spanish version of the Utrecht Work Engagement Scale (UWES) developed by Schaufeli et al. [32]. This consists of 17 items (e.g., “In my work I feel full of energy”). The responses presented a 7-point Likert-type format for all the items, with a range from 1 (totally disagree) to 7 (totally agree).

#### 2.3.3. Role Ambiguity

This dimension was measured using the Rizzo, House, and Lirtzman [65] role stress questionnaire in its Spanish version [66]. This dimension consists of 6 items (e.g., “I know the degree of autonomy of my work well”). The response scale is Likert-type, with 5 anchor points, ranging from 1 (totally disagree) to 5 (totally agree).

#### 2.3.4. Extra-Role Performance Behaviors

These behaviors were measured using the Goodman and Svyantek [67] questionnaire. The extra-role behavior dimension is made up of 3 items (e.g., “we help our colleagues with their work when they have to be absent”). A 7-point Likert-type response scale was used for all of them, ranging from 1 (strongly disagree) to 7 (strongly agree).

### 2.4. Data Analysis

Data were analyzed using SPSS 25 (IBM Corp., Armonk, NY, USA). After computing descriptive data, Cronbach’s alphas, and zero-order relationships between all constructs, mediation and moderation analyses were conducted (See Figure 1). Following the recommendations of Cristea et al. [68], a multi-step mediation analysis was used to test whether the effect of role ambiguity on extra-role behavior is mediated by engagement, the effect of which is further extended to reduce levels of engagement to an employee, which is one of the antecedents of extra-role behaviors. In addition, the model also includes leaders´ support behaviors as a moderator of the relationship between role ambiguity and employee engagement. Mediation and moderation analyses were conducted to estimate direct and indirect influence using the non-parametric bootstrapping procedure in the PROCESS package. The suggestion of Hayes [63] was followed by initially conducting a multi-step mediation analysis to find the effect of role ambiguity on extra-role behavior with the variables leader support and engagement as mediators 1 and 2, respectively (Model 6 in PROCESS). Subsequently, simple moderation analysis was carried out to identify if the leader’s support moderates the model (Model 7 in PROCESS).

Indirect and conditional influences were deemed significant if the 95% bias-corrected (BC) bootstrap confidence intervals (CI) based on 10,000 samples were not included. The fully standardized indirect effect (abcs; [69,70]) was used to calculate mediation effect sizes, with 95% baseline confidence intervals for BC. This measurement is based on the product of the betas for routes a and b, which provides us with the expected change in the dependent variable (e.g., extra-role performance behaviors) for each unit in which it varies in the predictor variable (e.g., role ambiguity) indirectly through the mediator (e.g., engagement). 

## 3. Results

### 3.1. Descriptive Data, Internal Consistencies, and Zero-Order Correlations 

Table 2 shows the descriptive data, internal consistencies, and correlations between the measures of this study. Participants’ average scores on engagement, personal recognition, and role-playing behaviors were higher than the center point on the scale. Participants’ mean scores on role ambiguity were lower than the center point on the scale. The internal consistencies of the scales ranged from 0.82 (Extra-role behavior) to 0.90 (Role ambiguity). The values found are similar to those found in previous studies that have used the same instruments. They all show strong correlations.

### 3.2. Testing the Mediation Model

Table 3 presents the results of the models contrasted through mediation. In the first model, role ambiguity was shown to be a significant predictor of extra-role performance behaviors. In the second model, it is observed how role ambiguity was a significant negative predictor of engagement. The third model shows how engagement was a significant mediator of the influence of role ambiguity on extra-role performance behavior. The fourth model informed about the negative influence of role ambiguity on leader support. The fifth model presented how leader support was a significant mediator of the influence of role ambiguity on extra-role performance behavior. In the sixth model presented, it is observed that all the predictor variables have a significant influence on the extra-role performance behavior, showing a decrease in the role ambiguity coefficient from −0.660 to −0.311 concerning the first model. 

Table 4 shows the general and indirect effects (EI). The results provide a significant mediation with a total EI of −0.349 (SE = 0.060, CI 95% BC from −0.473 to −0.234) with a high effect size (abcs = −0.255, CI 95% BC from −0.346 to −0.171). The rest of the specific indirect effects were also significant (IE1: TE = −0.186, SE = 0.043, CI 95% BC from −0.280 to −0.108, abcs = −0.136; IE2: TE = −0.134, SE = 0.048, CI 95% BC of −229. A −0.039, abcs = −0.098; IE3: TE = −0.028, SE = 0.012, CI 95% BC of −0.050 to −0.007, abcs = −0.020).

Table 5 shows how the effect of role ambiguity on engagement depends on the leader’s levels of support (interaction coefficient: role ambiguity x leader support = 0.079, SE = 0.037, *p* < 0.001). 

The results have demonstrated the negative influence of role ambiguity on extra-role performance behaviors (TE = −0.660, SE = 0.067, *p* <0.001). In the second hypothesis, the negative influence of role ambiguity on engagement was confirmed (TE = −0.438, SE = 0.038, *p* < 0.001). In the third hypothesis, the mediation model of engagement on the influence of role ambiguity is demonstrated (IE = −0.134, SE = 0.048, 95% BC CI of −0.229 to −0.039). 

The fourth hypothesis proposed the negative influence of role ambiguity on leader support, and this hypothesis has been confirmed (TE = −0.806, SE = 0.099, *p* < 0.001). In the fifth hypothesis, the mediation model of leader support on the influence of role ambiguity of extra-role performance behaviors has been shown (IE = −0.186, SE = 0.043, 95% BC CI of −0.280 to −0.208). 

Regarding moderated mediation, the sixth hypothesis establishes that role ambiguity on engagement is moderated by the degree of support from the leader support (TE = 0.079, SE = 0.037, *p* < 0.033). The seventh hypothesis of this paper proposed that the mediating process of engagement on the influence of role ambiguity on extra-role performance behaviors will depend on the support behaviors that the leader will carry out. The result of the moderate mediating model confirms this hypothesis (IE = −0.349, SE = 0.060, 95% BC CI of −0.473 to −0.234); it is possible to affirm that the degree of support by the leader influences the effect of role ambiguity in the intention to carry out additional performance behaviors in the workplace, through the influence on employee engagement.

## 4. Discussion

The main objective of the research study has been to analyze the moderating effect of support actions by the leader on the negative influence of role ambiguity on engagement and, in turn, on extra-role performance behaviors of employees. According to the results obtained, it can be concluded that our hypotheses have been supported. 

These results sustain the arguments defended by authors such as Orgambídez-Ramos et al. [18] and Pecino et al. [6], showing the negative influence of role ambiguity on support leadership and engagement. However, these results indicate that this negative influence of the effect of role ambiguity on engagement will be reduced depending on the degree of support used by the leader. When a leader shows support behaviors to his employees, it produces a strong buffering effect on stressors in the workplace [12,13,71], mainly regulating the effect of the stressor on engagement. Likewise, Britt et al. [60] found that the leader’s support behaviors in highly ambiguous situations produce positive employee results. Still, the results of the present manuscript show that this effect occurs in a double way: through a mediating effect and a moderating effect of the influence of role ambiguity on engagement. Confirming these hypotheses has theoretical and practical implications that are discussed below, along with limitations that could be used to develop additional research studies on these topics.

### 4.1. Theoretical Implications

To date, role ambiguity has been one of the most studied job demands, producing significant negative consequences in the work context [2,8,20]. However, a relevant contribution to the knowledge of the current study is that the demands are transformed depending on contextual factors in the public administration. From the recognized JD–R theory of Bakker and Demerouti [9], a link is established between the obstacle demand (role ambiguity), the behavior (extra-role behavior), and the emotional state (engagement). The benefice of leader support on role ambiguity influence happens in a double way: first, by the mediation effect of leader support on the effects of the ambiguity, and second, by the mediating moderation model. This confirms that in the face of an increase in support behaviors by the leader (contextual resources), the negative effect of role ambiguity is reduced. It facilitates the appearance of a high level of engagement, in turn of extra-role behaviors. 

In addition, the results of the present study also help to clarify the possibility of a variable acting as a mediator and a moderator at the same time in a relationship model, increasing its total effect on an outcome variable, which would support the proposal made by authors such as Chmura Kraemer et al. [62] and Kenny [64].

For all this, it is possible to confirm that a public workplace where the leader’s support behaviors are developed produces high motivation and performance behaviors beyond what is formally established in the public organization, developing a positive work context and avoiding rigidity and passivity of the public sector.

### 4.2. Practical Implications

The training of supporting a leader’s behavior is fundamental to fulfilling strategic and institutional objectives in public administration [58,72,73,74]. Although the leader’s support has already been shown to provide important advantages, such as higher productivity levels [75], results such as those provided in this paper also show the importance of facing contexts with high levels of stress in public administration. The existence of programs to develop the professional skills of leaders acquires a unique strategic dimension centered on the patterns of transformative behavior in organizations. However, it is necessary to contextualize the scope of application correctly. These procedures for improving the skills of leaders imply an increase in the resources of the employees that link them to a greater extent with the organization at the same time that they will increase their intention to carry out behaviors beyond what is stipulated in their functions [76]. However, despite the possibility of protection that the support provides, the need to further clarify the functions and tasks that a person has to develop is an objective that the public administration should not neglect; even if a leader is willing to support employees, the benefit of these actions will be more significant if it is also possible to improve a correct definition of the positions.

### 4.3. Limitations and Future Investigations

As with different studies, the present one has limitations to be reported and offers expansion for future research. First, there are limitations regarding the method used since the information was collected through online questionnaires (self-reports), so they could be affected by the variance of the common method [77,78]. Future research would be advisable to attribute more of the variables considered as intersubjective responses at the team level to the measurement method. A key element that would guarantee the validity of the data would be the use of complementary instruments such as direct observation, interviews with employees and superiors, or the collection of objective data to determine productivity and performance. The sample gives the second limitation. This is very specific and is limited to public personnel in Spain. Therefore, the results cannot be generalized to another type of organization. It would be very interesting in future investigations to compare samples between public and private administrations [53,78], or to expand the number of organizations from different public bodies (state, regional, and local), or to carry out cross-cultural studies. Third, the research design is cross-sectional and provides less information than other types of studies. Multilevel and longitudinal studies are necessary to analyze the effects of group membership and the evolution of the variables studied to improve work teams’ attitudes, behaviors, and group performance, or examine how these variables affect performance and productivity, and, indirectly, organizational behaviors such as cordiality or citizenship attitudes.

## 5. Conclusions

This study provides a challenge for future research, since the contributions of contextual and team-level resources to the motivational processes implicit in the JD–R model have not been fully explored. Our study provides a key tool to help explain how leader support can generate higher levels of engagement and extra-role behaviors in highly ambiguous situations in public organizations, leading to this obstacle demand becoming positive. This would be a new step in understanding demand and resource theory. Finally, it is evident in this study that leaders must be aware that their support is essential for those who serve in the organization, helping employees to deal with situations of role ambiguity and improving healthy and competitive organizational efficiency in the public sector.

## Figures and Tables

**Table 1:** Demographic data of the sample.

Factor	Total Sample
Gender	
N	315
% Male	51.6
% Female	48.4
Age	
N	315
% under 36 years old	1.8
% between 36 and 45 years old	14.1
% between 46 and 55 years old	64
% over 55 years	20.1
Academic level	
N	315
% Elementary or High School	10.3
% Bachelor’s degree	23
% Graduate	55.5
% Master’s degree	10.1
% PhD	1.1
Employment status	
N	315
% Public officials	92.9
% Ordinary employees	7.1
Job positions	
N	315
Managers	7.1
Pre-managers	15.9
Operating	77
Working hours	
N	315
Morning shift	81.9
Afternoon shift	18.1

**Table 2:** Descriptive data, internal consistencies, and correlations.

	M	SD	α	2	3	4
1. Role Ambiguity	1.95	0.81	0.90	−0.608 **	−0.416 **	−0.483 **
2. Engagement	4.31	1.34	0.86		0.465 **	0.487 **
3. Leader Support	3.94	1.62	0.88			0.515 **
4. Extra-Role Behavior	4.70	1.16	0.82			

** *p* > 0.001.

**Table 3:** Results from the regression analyses examining the mediator model of the effect of role ambiguity (x) on extra-role performance behavior (y) through leader support (m1) and engagement.

	Coefficient	SE	*p*
Model 1 (extra-role performance behaviors) Total Effect			
X (Role ambiguity)	−0.660	0.067	<0.001
Constant	6.000	0.144	<0.001
R^2^ = 0.373			
F = 95.444, *p* ≤ 0.001			
Model 2 (engagement)			
X (Role ambiguity)	−0.988	0.072	<0.001
Constant	6.248	115	<0.001
R^2^ = 0.370			
F = 184.157, *p* ≤ 0.001			
Model 3 (extra-role performance behaviors)			
X (Role ambiguity)	−0.405	0.082	<0.001
M1 (Engagement)	0.257	0.050	<0.001
Constant	4.393	0.345	<0.001
R^2^ = 297			
F = 64.552, *p* ≤ 0.001			
Model 4 (leader support)			
X (Role ambiguity)	−0.806	0.099	<0.001
Constant	5.526	0.212	<0.001
R^2^ = 0.173			
F = 65.543, *p* ≤ 0.001			
Model 5 (extra-role performance behaviors)			
X (Role ambiguity)	−0.445	0.068	<0.001
M2 (Leader support)	0.266	0.035	<0.001
Constant	4.531	0.236	<0.001
R^2^ = 0.351			
F = 84.660, *p* ≤ 0.001			
Model 6 (extra-role performance behaviors)			
X (Role ambiguity)	−0.311	0.078	<0.001
M1 (Engagement)	0.165	0.049	<0.001
M2 (Leader support)	0.231	0.036	<0.001
Constant	3.694	0.343	<0.001
R^2^ = 0.151			
F = 61.195, *p* ≤ 0.001			

**Table 4:** Indirect effects of the serial multiple mediator model of the effect of role ambiguity (x) on extra-role behavior (Y) through engagement (M1) and leader support (M2).

			BootstrappingBC 95% CI	
	Coefficient	SE	Lower	Upper
Overall indirect effect	−0.349	0.060	−0.473	−0.234
IE1:X ≥ M1 ≥ Y	−0.134	0.048	−0.229	−0.039
IE2:X ≥ M2 ≥ Y	−0.186	0.043	−0.280	−0.108
IE3: X ≥ M1 ≥ M2 ≥ Y	−0.028	0.012	−0.050	−0.007

**Table 5:** Results of regression analysis examining the moderation of the influence of role ambiguity on employee engagement by leader support.

Antecedent	Coefficient	SE	*p*
X (Role ambiguity)	−0.988	0.072	<0.001
W (Leader support)	0.054	0.084	0.517
XxW	0.079	0.037	0.033
Constant	6.248	0.115	<0.001
R^2^ = 0.432 F = 79.138, *p* = 0.000			

## Data Availability

The data that support the findings of this study are available on request from the corresponding author (P.A.D.-F.). The data are not publicly available due to their containing information that could compromise the privacy of research participants.
